# Epidemiologic Features and Outcomes of Caustic Ingestions; a 10-Year Cross-Sectional Study

**Published:** 2017-02-02

**Authors:** Athena Alipour Faz, Fahimeh Arsan, Hassan Peyvandi, Mahbobeh Oroei, Omid shafagh, Maryam Peyvandi, Maryam Yousefi

**Affiliations:** 1 Clinical Research Development Center, Loghman Hakim Hospital, Shahid Beheshti University of Medical Sciences, Tehran, Iran; 2 General Surgery Department, Loghman Hakim Hospital, Shahid Beheshti University of Medical Sciences, Tehran, Iran.; 3 Hearing Disorders Research Center, Loghman Hakim Hospital, Shahid Beheshti University of Medical Sciences, Tehran, Iran.

**Keywords:** Caustics, epidemiology, toxicology, suicide, attempted, patient outcome assessment

## Abstract

**Introduction:**

Caustic ingestions are among the most prevalent causes of toxic exposure. The present 10-year survey aimed to evaluate the epidemiologic features and outcomes of caustic ingestion cases presenting to emergency department.

**Methods:**

This is a retrospective cross-sectional study on patients who were admitted to a referral toxicology center during 2004 to 2014, following caustic ingestion. Baseline characteristics, presenting chief complaint, severity of mucosal injury, complications, imaging and laboratory findings as well as outcomes (need for ICU admission, need for surgery, mortality) were recorded, reviewing patients’ medical profile, and analyzed using SPSS 22.

**Results:**

348 patients with mean age of 37.76 ± 17.62 years were studied (55.6% male). The mean amount of ingested caustic agent was 106.69 ± 100.24 mL (59.2 % intentional). Intentional ingestions (p < 0.0001), acidic substance (p = 0.054), and higher volume of ingestion (p = 0.021) were significantly associated with higher severity of mucosal damage. 28 (8%) cases had died, 53 (15.2%) were admitted to ICU, and 115 (33%) cases underwent surgery.

**Conclusion:**

It seems that, suicidal intention, higher grade of mucosal injury, higher volume of ingestion, lower level of consciousness, lower serum pH, and higher respiratory rate are among the most important predictors of need for ICU admission, need for surgery, and mortality.

## Introduction

Caustic ingestions are among the most prevalent causes of toxic exposure. The most commonly ingested caustics are acidic or alkaline substances (1). Ingestion of cleaning substances account for more than 200,000 annual exposures reported to the United States poison control center (2-4). 10% of adults referring with caustic ingestions expire and 1-2% of ingestions lead to stricture formation (5). Children younger than 5 years account for approximately 80% of caustic ingestion cases that typically occur accidentally. In adolescents, caustic ingestion cases are generally in an attempt to commit suicide and they present in more severe conditions (6, 7). Dysphagia, odynophagia, drooling, vomiting, stridor, dyspnea, and oropharyngeal lesions are among the most important complaints of patients. 

Caustic ingestion can induce acute and chronic injuries and treatment in both stages is important. The severity of tissue injury in the acute phase depends on multiple factors including the type of substance, its concentration, the ingested amount, and the duration of contact (8). In the chronic stage, treatments concentrate on preventing development of strictures and improving function of the esophagus (9, 10). Loghman-Hakim toxicology center is one of the big referral centers for these injuries in Iran capital, Tehran. Therefore, the present survey aimed to evaluate the epidemiologic features and outcomes of caustic ingestion cases presenting to this center during a 10-year period.

## Methods

***Study design and setting***

This is a retrospective cross-sectional study on patients who were hospitalized in Loghman-Hakim Hospital, Tehran, Iran, during 2004 to 2014, following ingestion of caustic substances. The study protocol was evaluated and approved by the Ethics Committee of Shahid Beheshti University of Medical Sciences. All the information gathered was considered confidential and was used anonymously throughout the study. Researchers adhered to all ethical principles presented in declaration of Helsinki during the study period.

***Participants***

All patients who were admitted to the mentioned toxicology center following ingestion of caustic agents were enrolled using census sampling. There was not any sex, age, ethnic or etc. limitation. Loghman-Hakim Hospital is one of the main referral centers for poisoning and intoxication emergencies in Iran and patients from all over the country are referred to this hospital. Therefore, this center hosts a diverse population of patients with various problems that can be considered as a national representative sample.

***Data gathering***

Patients’ baseline characteristics (age, sex, clinical findings, intent of ingestion , type of caustic substance (acidic or alkaline), amount of ingested caustic (as reported by the patients or the patients’ family members), time to hospital, duration of hospitalization, duration of ICU admission), presenting vital signs (systolic blood pressure, respiratory rate, pulse rate, Glasgow coma scale), presenting chief complaint (vomiting, dyspnea, drooling, etc.) severity of mucosal injury on esophagogastroduodenoscopy (EGD), gastrointestinal complications (perforation, strictures and fistula formation), systemic complication (acute renal insufficiency and acute hepatic failure), chest x ray findings, pathology findings, and laboratory findings (sodium, potassium, blood urea nitrogen, creatinine, aspartate aminotransferase (AST), alanine aminotransferase (ALT), blood gas analysis (pH, HCO3, PCO2)) as well as outcomes (need for ICU admission, need for surgery, mortality) were recorded reviewing patients’ medical profile. 

The severity of mucosal injuries was graded based on Zargar's modified endoscopic classification (11). Patients with grade I burns are just monitored for 24 to 48 hours while patients with grade 2 and 3 undergo exploratory laparotomy (12-15). 

Acute renal insufficiency was defined as glomerular filtration Rate < 60 mL/min/1.73 M^2^ and acute hepatic failure as increased liver function tests to > 3 times the normal upper limit. A trained medical doctor was responsible for data gathering. 

***Statistical analysis***

Data were analyzed using SPSS software for windows version 22. The quantitative data were described by mean ± standard deviation and qualitative variables were presented using frequency and percentage. The associations of baseline, clinical, and laboratory variables with need for surgery, need for ICU admission, and mortality were estimated using appropriate statistical tests such as chi square, Fisher’s exact, t test, one way ANOVA, or non-parametric tests. The significance level was considered P < 0.05. 

## Results

***Baseline Characteristics***

348 patients with mean age of 37.76 ± 17.62 years (3 – 87) were studied (55.6% male). Table 1 shows the baseline characteristics of studied patients. 

The mean amount of ingested caustic agent was 106.69 ± 100.24 mL (10 – 500). Hydrochloric acid was the most commonly (69.8%) ingested acidic substance, which was followed by sulfuric acid (19.5%). The most common ingested alkaline was lye (46.3%). Among intentional cases, 115 (56.1%) patients had used alkaline material and 90 (43.9%) acidic ones (p = 0.812).

***Laboratory findings***

Table 2 summarizes the laboratory findings of studied patients. 21.7% hypernatremia, 5.1% hyperkalemia, 3.9% abnormal creatinine (≥ 1.6 mg/dl), and 8.2% abnormal liver function test were among the most important laboratory findings. Serum pH revealed 33.1% acidosis and 15.2% alkalosis.

***Chest X ray findings***

1 (0.5%) case of hemothorax, 2 (1.1%) sub-diaphragmatic free gas, 5 (2.7%) airway edema, 2 (1.1%) reticulonodular changes, and 2 (1.1%) aortic ectasia cases were among the most important chest X-ray findings, which were recorded for 186 cases. 

***Endoscopic findings***

The findings of EGD were available for 313 patients. The severity of esophageal mucosal damage based on Zargar's modified endoscopic classification was normal in 64 (20.4), grade I in 133 (42.5%), grade IIa in 37 (11.8%), grade IIb in 16 (5.1%), and grade IIIa in 63 (20.1%) cases. These measures for gastric mucosal damage were 72 (25.9) normal cases, 72 (25.9%) grade I, 62 (22.3%) grade IIa, 32 (11.5%) IIb, 39 (14.0%) grade III, and 1 (0.4%) grade IV. Intentional ingestions (p < 0.0001), acidic substance (p = 0.054), and higher volume of ingestion (p = 0.021) were significantly associated with higher severity of mucosal damage. 

***Pathology findings***

The results of pathological assessment were available for 49 (14.1%) cases, which revealed grade I injury in 13 (3.7%), grade II in 23 (6.6%), and grade III in 13 (3.7%) cases. 

***Outcomes***

28 (8%) cases had died (2.9% in < 24 hours of admission) and 28 (8%) cases were discharged against medical advice. The mean duration of hospital stay was 5.76 ± 6.76 (1 – 50) days. 3 (0.9%) acute renal insufficiency, 5 (1.4%) gastrointestinal perforation, 33 (9.5%) stricture formation, and 3 (0.9%) fistula formation cases were among the most important complications in the present series. 

***Need for ICU admission***

53 (15.2%) were admitted to the ICU for 6.32 ± 6.12 days (1 – 41). Table 3 shows the correlation of different demographic, clinical, and endoscopic variables with need for ICU admission. There were a significant correlation between suicidal intention (p < 0.0001), higher grade of mucosal injury on EGD (p < 0.0001), volume of ingestion (p < 0.0001), delayed admission (p = 0.033), level of consciousness on admission (p = 0.001), serum pH on admission (p = 0.003), respiratory rate on admission (p = 0.035), and dyspnea (p < 0.0001) with need for ICU admission. 

***Need for surgery***

115 (33%) cases were in need of surgical interventions and underwent surgery. Acidic caustic agent (p = 0.027), suicidal intention (p < 0.0001), higher grade of mucosal injury on EGD (p < 0.0001), volume of ingestion (p = 0.010), level of consciousness on admission (p = 0.0001), serum pH on admission (p = 0.001), respiratory rate on admission (p < 0.0001), and dyspnea (p = 0.001) were significantly associated with need for surgery. 

***Mortality***

Table 4 shows the correlation of different demographic, clinical, and endoscopic variables with mortality. There were a significant correlation between mean age (p = 0.032), suicidal intention (p = 0.002), higher grade of mucosal injury on EGD (p < 0.0001), volume of ingestion (p = 0.001), level of consciousness on admission (p < 0.001), serum pH on admission (p < 0.001), and respiratory rate on admission (p < 0.001) with mortality. 

## Discussion

Based on the findings of present study, most cases of caustic ingestion were between 18 – 35 year old (42%), with 80% female to male ratio, 79% alkaline to acid ratio, 50% unintentional to intentional ratio, and normal to grade I mucosal injury.

 Suicidal intention, higher grade of mucosal injury, higher volume of ingestion, lower level of consciousness, lower serum pH, and higher respiratory rate were significantly correlated with need for ICU admission, need for surgery, and mortality.

The sex and age distribution of the study participants were compatible with the results of Paudyal et al. in Nepal (16) and unmatched with the findings of Istanbul study which declared the 3:1 female to male ratio of caustic ingestion (17). In this survey, alkaline substances had a higher prevalence compared to acidic solutions. Arévalo‐Silva et al. also reported the predominance of alkaline substance in their study (1). The reason for ingestion was also found to be intentional in the majority of patients, which was associated with severe injuries. These findings were similar to Cheng et al. study (18).

**Table1:** Baseline characteristics of studied patients

**Variable**	**Value**
**Age (year)**	
< 18	25 (7.3)
18 – 35	144 (42)
35 – 50	97 (28.3)
50 – 65	39 (11.4)
≥ 65	38 (11.1)
**Sex**	
Male	193 (55.6)
Female	154 (44.4)
**Caustic type**	
Acidic	188 (54.0)
Alkaline	149 (42.8)
Unknown	11 (3.2)
**Intention**	
Suicidal	206 (59.2)
Accidental	105 (30.2)
Unknown	37 (10.6)
**Time to reach ED (hour)**	
< 6	253 (81.87)
6 - 12	29 (9.38)
>12	27 (8.73)
**Presenting signs**	
Vomiting	164 (47.8)
Hematemesis	145 (42.3)
Dysphagia	88 (25.7)
Drooling	68 (19.8)
Oropharyngeal lesions	63 (18.4)
Dyspnea	50 (14.6)
**History of suicidal attempt (n =78)**	
Yes	47 (60.3)
No	31 (39.7)
**History of psychiatric disorders (n =105)**	
Yes	72 (68.6)
No	33 (31.4)
**Vital signs on admission**	
Systolic blood pressure (mmHg)	119.76±19.26
Pulse rate (/minutes)	86.29±14.72
Respiratory rate (/minute)	19.08±4.98
Glasgow coma scale	14.75±0.92

Data were presented as mean ± standard deviation or number and percentage.

**Table 2:** Laboratory findings of the studied patients at the time of admission to emergency department

**Variable**	**Number (%)**
**Sodium (mEq/dL)**	
< 135	12 (3.6)
135 – 145	248 (74.7)
> 145	72 (21.7)
**Potassium (mEq/dL)**	
< 3.5	10 (3.0)
3.5 – 5.5	305 (91.9)
> 5.5	17 (5.1)
**Blood urea nitrogen (mg/dl)**	
< 20	48 (14.5)
≥ 20	284 (85.5)
**Creatinine (mg/dl)**	
< 1.6	326 (96.1)
≥ 1.6	13 (3.9)
**Aspartate aminotransferase (U/L)**	
< 40	56 (91.8)
≥ 40	5 (8.2)
**Alanine aminotransferase (U/L)**	
< 40	57 (95.0)
≥ 40	3 (5.0)
**pH**	
<7.35	85 (33.1)
7.35 -7.45	133 (51.8)
>7.45	69 (15.2)
**HCO3 (mmol/L)**	
< 22	128 (50.6)
22 – 26	75 (29.6)
≥ 26	50 (19.8)
**PCO2 (mmHg)**	
<35	99 (28.4)
35 – 45	104 (40.8)
≥ 45	52 (20.4)

**Table 3:** Correlation of demographic, clinical, laboratory, and endoscopic variables with need for ICU admission

**Variable**	**ICU admission**		**P value**
	**No**	**Yes**	
**Sex**			
Male	158 (81.9)	35 (18.1)	0.101
Female	136 (88.3)	18 (11.7)	
**Type of caustic agent**			
Alkaline	164 (87.2)	24 (12.8)	0.114
Acidic	122 (81.9)	27 (18.1)	
**Intention**			
Accidental	100 (95.2)	5 (4.8)	< 0.001
Suicidal	165 (80.1)	41 (19.9)	
**History of psychiatric disease**			
Yes	53 (73.6)	19 (26.4)	0.068
No	30 (90.9)	3 (9.1)	
**Esophageal injury (endoscopic)**			
Grade I	127 (95.5)	6 (4.5)	< 0.001
Grade IIa	31 (83.8)	6 (16.2)	
Grade IIb	13 (81.3)	3 (18.8)	
Grade IIIa	34 (54.0)	29 (46)	
**Gastric injury (endoscopic)**			
Grade I	68 (94.4)	4 (5.6)	< 0.001
Grade IIa	56 (90.3)	6 (9.7)	
Grade IIb	23 (71.9)	9 (28.1)	
Grade III	27 (69.2)	12 (30.8)	
Grade IV	0 (0.0)	1 (100)	
**Serum pH on admission**			
< 7.35	62 (72.9)	23 (27.1)	0.003
7.35 – 7.45	120 (90.2)	13 (9.8)	
> 7.45	33 (84.6)	6 (15.4)	
**Dyspnea**			
Yes	18 (64)	35 (36)	< 0.001
No	258 (88.1)	35 (11.9	
**Mean age (year)**	37.19 ± 17.86	40.92 ± 16.00	0.160
**Delayed admission (hour)**	3.37 ± 3.98	4.76 ± 5.1	0.033
**Volume of ingestion **	96.16 ± 90.03	165.57 ± 131.03	< 0.001
**Glasgow coma scale on admission**	14.85 ± 0.55	13.91 ± 2.21	0.001
**Respiratory rate (/minute)**	18.85 ± 4.57	20.52 ± 6.91	0.035

Data were presented as mean ± standard deviation or number and percentage.

**Table 4:** Correlation of demographic, clinical, laboratory, and endoscopic variables with mortality

**Variable**	**Mortality**		**P value**
	**No**	**Yes**	
**Sex**			
Male	179 (92.7)	14 (7.3)	0.557
Female	410 (90.9)	14 (9.1)	
**Type of caustic agent**			
Alkaline	173 (92.0)	15 (8.0)	0.844
Acidic	136 (91.3)	13 (8.7)	
**Intention**			
Accidental	103 (98.1)	2 (1.9)	0.002
Suicidal	182 (88.3)	24 (11.7)	
**History of psychiatric disease**			
Yes	61 (84.7)	11 (15.3)	0.771
No	29 (87.9)	4 (12.1)	
**Esophageal injury (endoscopic)**			
Grade I	130 (97.7)	3 (2.3)	< 0.0001
Grade IIa	36 (97.3)	1 (2.7)	
Grade IIb	14 (87.5)	2 (12.5)	
Grade IIIa	48 (76.2)	15 (23.8)	
**Gastric injury (endoscopic)**			
Grade I	72 (100)	0 (0.0)	< 0.0001
Grade IIa	57 (91.9)	5 (8.1)	
Grade IIb	31 (96.9)	1 (3.1)	
Grade III	27 (69.2)	12 (30.8)	
Grade IV	0 (0.0)	1 (100)	
**Serum pH on admission**			
< 7.35	66 (77.6)	19 (22.4)	< 0.001
7.35 – 7.45	128 (96.2)	5 (3.8)	
> 7.45	38 (97.4)	1 (2.6)	
**Delayed admission (hour)**	3.59 ± 4.28	3.48 ± 2.88	0.899
**Mean age (year)**	37.15 ± 17.36	44.61 ± 19.37	0.032
**Volume of ingestion **	101.68 ± 95.73	178.16 ± 134.53	0.001
**Glasgow coma scale on admission**	14.85 ± 0.62	13.17 ± 2.56	< 0.001
**Respiratory rate (/minute)**	18.71 ± 4.41	23.68 ± 8.34	< 0.001

Data were presented as mean ± standard deviation or number and percentage.

Based on the endoscopic findings, most patients (42.5%) had grade I mucosal injury in their esophagus and stomach, which was congruent with the findings of Arévalo‐Silva et al. (1). 

There are numerous studies on characteristics of caustic ingestions and predictors of their poor outcome. Endoscopic grading of mucosal damage is reported as a helpful tool in this regard (19). Cheng and their colleagues in the study of adult caustic ingestion showed the correlation of grade IIIb mucosal damage with higher morbidity rate (18). 

Delayed admission was associated with higher need for ICU admission in the present study. 

Yeganeh and their colleagues showed that early admission can reduce the mortality rate of corrosive ingestion (20). 

Older age had been associated with poorer clinical outcome in patients with caustic ingestion (21). Caustic ingestion with suicidal intention was correlated with higher rate of mortality and poorer outcome (22). 

In addition, acid ingestions were associated with severe complication and higher mortality rate than alkaline in some studies (22-24). 

Lower serum pH is reported as an indicator of severe injury in blood gas analysis of caustic ingestion cases (25).

In this survey, 42.5% of patients were discharged from hospital after two days of hospitalization in observation unit and 15.2% of the patients were admitted to the ICU. One third of patients needed surgery and underwent laparotomy, gastrotomy, biopsy, stent placement, and feeding jejunostomy. 

Collectively, based on the study findings, it seems that the main predictor of poor outcome in caustic cases is intention type. If caustic ingestion happens with suicidal attempt, it would be linked with higher volume, high potent solution, and delayed admission, all of which are indicators of poorer outcomes. 

Planning for preventive measures may seem ineffective in intentional cases, but it would be very helpful in decreasing the unintentional ones. Using cleaning and detergent agents with safe formula and keeping them away from children could be considered for minimizing the severity and number of unintentional cases.

***Limitations***

This is a retrospective cross-sectional study with its natural limitations that missing data is among the most important of them. 

## Conclusion:

It seems that, suicidal intention, higher grade of mucosal injury, higher volume of ingestion, lower level of consciousness, lower serum pH, and higher respiratory rate are among the most important predictors of need for ICU admission, need for surgery, and mortality.
